# A bibliometric analysis of research trends in mesenchymal stem cell therapy for neonatal bronchopulmonary dysplasia: 2004–2024

**DOI:** 10.3389/fped.2025.1558301

**Published:** 2025-06-03

**Authors:** Lu Bai, Yue Xin

**Affiliations:** Department of Pediatrics, Tianjin Medical University General Hospital, Tianjin, China

**Keywords:** bibliometric analysis, bronchopulmonary dysplasia, mesenchymal stem cells, research trends, citation analysis, international collaboration

## Abstract

**Introduction:**

Bronchopulmonary dysplasia (BPD) is a chronic lung disease predominantly affecting preterm infants, often requiring mechanical ventilation and supplemental oxygen. The pathogenesis of BPD involves a combination of genetic susceptibility and environmental insults, such as oxidative stress and mechanical ventilation. Mesenchymal stem cells (MSCs) have emerged as a promising therapeutic option for BPD due to their immunomodulatory, anti-inflammatory, and regenerative properties. This study aims to perform a bibliometric analysis of the publication landscape surrounding MSC therapy for BPD to identify research trends, collaborative networks, influential research clusters, and emerging research frontiers from 2004 to 2024.

**Methods:**

A bibliometric analysis was conducted using the Web of Science Core Collection (WoSCC) as the primary database due to its comprehensive citation indexing and standardized metadata. To ensure data integrity, we included publications from January 2004 (when the first relevant MSC studies for BPD began appearing) to November 2024. The search query combined terms related to BPD and MSCs, focusing on English-language articles and reviews. After retrieval, data were cleaned through duplicate removal and relevance verification processes. Quantitative analysis was performed on publication counts, authors, journals, institutions, and countries. Visual analysis tools, VOSviewer ( 1) and CiteSpace ( 2), were employed to map collaboration networks and identify research clusters through co-citation and co-occurrence analyses. Statistical validation of bibliometric distributions was conducted using Bradford's law and Price's law. Citation metrics were normalized by publication year to account for citation accumulation bias.

**Results:**

A total of 353 publications were analyzed, including 216 articles and 137 reviews, from 555 institutions across 35 countries. Time-series analysis revealed a significant acceleration in publication output after 2015 (*p* < 0.01), with a compound annual growth rate of 18.2%. The United States was the leading contributor (131 publications, 37.1%), followed by China (72 publications, 20.4%) and Canada (54 publications, 15.3%). Network analysis identified five distinct collaborative clusters, with limited cross-cluster collaboration. Citation analysis, normalized for publication age, revealed that the American Journal of Respiratory and Critical Care Medicine had the highest field-weighted citation impact (3.8). Keyword co-occurrence analysis demonstrated a significant shift from whole-cell therapies to extracellular vesicle research after 2018, with “microvesicles” and “exosomes” emerging as high-intensity burst terms (burst strength >5.0). The co-citation analysis identified three primary research clusters: stem cell therapy mechanisms (42.3% of citations), respiratory physiology and pathology (38.1%), and clinical neonatology (19.6%).

**Conclusion:**

This bibliometric analysis maps the evolving landscape of MSC therapy research for BPD over the past two decades, revealing distinct research clusters with limited cross-disciplinary integration. Our findings demonstrate a clear shift from whole-cell MSC investigations toward MSC-derived exosomes as a cell-free therapeutic approach, particularly since 2018. Despite the growing body of preclinical evidence, visualization of publication patterns reveals significant gaps between laboratory findings and clinical applications, with only 8.2% of publications reporting clinical outcomes. The analysis further highlights geographical imbalances in research contributions and collaborative networks, suggesting opportunities for broader international engagement. These findings provide a foundation for directing future research efforts toward addressing knowledge gaps, particularly in understanding precise mechanisms of action and establishing standardized clinical protocols.

## Introduction

Bibliometric analysis is a quantitative approach to evaluating scholarly literature that employs statistical methods to identify patterns, trends, and relationships within a body of published research (1). Unlike traditional literature reviews or meta-analyses that focus on aggregating research findings, bibliometric analyses map the structure and evolution of a research field by examining publication metadata, citation patterns, and collaboration networks (2). This approach is particularly valuable for rapidly evolving fields where understanding research landscapes can inform strategic research directions and identify knowledge gaps. Modern bibliometric methodologies utilize advanced analytical tools such as those described by Aria and Cuccurullo (3) to provide comprehensive science mapping analyses.

Bronchopulmonary dysplasia (BPD) represents a significant challenge in neonatal medicine. Since 1967, when Northway first characterized BPD as a chronic lung condition linked to respirator therapy in preterm infants (4), its definition has evolved alongside advances in neonatal care. Over the past two decades, medical progress has significantly improved survival rates for preterm infants, particularly those with very low or extremely low birth weights. However, these infants remain at high risk for immature lung development, making them vulnerable to inflammatory insults, mechanical ventilation, hyperoxia, and other factors. Consequently, the incidence of BPD continues to rise (5). Recent therapeutic advancements, including prenatal glucocorticoids, pulmonary surfactant (PS) therapy, and non-invasive ventilation, have mitigated some risks, but the condition persists, with mild forms of BPD displaying distinctive pathological features, such as alveolar simplification, pulmonary microvascular dysplasia, and thickened alveolar septa (6).

BPD is a multifactorial condition, influenced by genetic susceptibility and a variety of prenatal and postnatal insults. Key factors include oxidative stress from hyperoxia, ventilator-induced injuries, and intrauterine inflammation (e.g., chorioamnionitis), which exacerbate lung damage and impair reparative mechanisms. The long-term consequences of BPD extend into childhood and adulthood, increasing the risks of chronic respiratory diseases such as wheezing, recurrent infections, chronic obstructive pulmonary disease (COPD), and cardiovascular disorders, alongside neurodevelopmental delays (7). Current interventions, including exogenous PS, tailored ventilator strategies, caffeine therapy, and anti-inflammatory agents such as corticosteroids, often achieve limited success and carry significant adverse effects. These limitations have driven the exploration of regenerative approaches, with mesenchymal stem cell (MSC) therapy emerging as a promising candidate for BPD management.

MSCs were first characterized over five decades ago by McCulloch and Till (8) and have gained significant attention for their therapeutic potential in various conditions, including BPD. These multipotent cells can be isolated from diverse sources, including bone marrow, adipose tissue, umbilical cord blood, and Wharton's jelly, offering practical advantages for clinical applications. The International Society for Cellular Therapy defines MSCs by specific criteria, including plastic adherence, expression of surface markers (CD105, CD73, CD90), absence of hematopoietic markers (CD45, CD34), and differentiation potential into mesodermal lineages (9). In BPD, MSCs appear to exert therapeutic effects primarily through paracrine mechanisms rather than direct engraftment, releasing bioactive factors that modulate inflammation, enhance tissue repair, and promote angiogenesis (10, 11). As highlighted by Thébaud (12), MSCs represent a promising therapeutic strategy for addressing the challenges of extreme prematurity and its associated complications.

Recent publications suggest a growing interest in MSC-derived extracellular vesicles (EVs), including exosomes and microvesicles, as potential cell-free alternatives to whole-cell therapy for BPD. This trend is supported by studies from Sdrimas and Kourembanas (13), who describe MSC microvesicles as a new paradigm for cell-free therapy. The field has evolved from preclinical investigations to early-phase clinical trials, with several publications reporting safety and preliminary efficacy data. Chang et al. (10, 11) have conducted pivotal phase I dose-escalation clinical trials of MSC therapy for BPD, demonstrating safety and potential efficacy. Subsequently, Ahn et al. (14, 15) have published two-year follow-up outcomes of these trials and conducted randomized controlled phase II trials, further advancing our understanding of clinical applications for MSC therapy in preterm infants with BPD. Additionally, Tung et al. (15) have explored the therapeutic potential of the stem cell secretome in neonatal diseases, highlighting the evolving focus on cell-free approaches. However, the landscape of this rapidly evolving field remains unclear, with limited systematic mapping of research networks, influential studies, and emerging trends.

To address this knowledge gap, we conducted a comprehensive bibliometric analysis of the MSC therapy for BPD literature published between 2004 and 2024. The primary objectives of this study were to: (1) map the temporal evolution of publication patterns and research foci; (2) identify influential authors, institutions, and countries driving the field; (3) characterize collaborative networks and potential silos; (4) visualize research clusters and emerging frontiers; and (5) highlight knowledge gaps where future research efforts may be directed. This analysis aims to provide valuable insights for researchers, funding agencies, and policymakers interested in advancing MSC-based therapies for BPD. Our approach is informed by established science studies methodologies as described by Hess (16), emphasizing the importance of understanding the social context of scientific knowledge production.

## Materials and methods

### Bibliometric analysis approach

Bibliometric analysis offers several advantages over traditional literature reviews for mapping research landscapes. Unlike narrative reviews that may be influenced by subjective selection biases, bibliometric approaches employ quantitative methods to analyze publication metadata, identifying patterns that might not be apparent through conventional literature synthesis. This approach is particularly valuable for examining the structural characteristics of a research field, including collaborative networks, citation patterns, and thematic evolution over time.

### Data sources and search strategy

The Web of Science Core Collection (WoSCC) served as the primary database for this bibliometric analysis. WoSCC was selected for its comprehensive coverage of peer-reviewed literature, standardized metadata, and robust citation tracking capabilities, as highlighted by Zhu and Liu (17). While databases such as PubMed offer broader coverage of biomedical literature, WoSCC provides more complete citation data necessary for co-citation analyses. Indexing was restricted to the Social Sciences Citation Index (SSCI) and Science Citation Index Expanded (SCI-EXPANDED) to ensure the inclusion of high-quality academic literature (17).

The search period was defined as January 2004 to November 2024, with the starting date corresponding to the emergence of the first publications exploring MSC applications in BPD-related models. This 20-year timeframe allows for comprehensive tracking of the field's evolution from early conceptual studies through preclinical investigations to clinical translation. All data were retrieved on 5 November 2024, at 11:01:52 GMT+0800 (CST), ensuring the most up-to-date results from the database.

The search strategy employed Medical Subject Headings (MeSH) terms and related keywords to capture comprehensive data on bronchopulmonary dysplasia (BPD) and mesenchymal stem cells (MSCs). The final search string was formulated as follows:

• **Query:** TS = (Bronchopulmonary Dysplasia OR Dysplasia, Bronchopulmonary OR Ventilator-Induced Lung Injuries OR Ventilator Induced Lung Injury) AND TS = (Mesenchymal Stem Cells OR Mesenchymal Stromal Cells OR Mesenchymal Progenitor Cells OR Wharton's Jelly Cells OR Adipose-Derived Mesenchymal Stromal Cells OR Multipotent Bone Marrow Stromal Cells).

**Figure 1:**
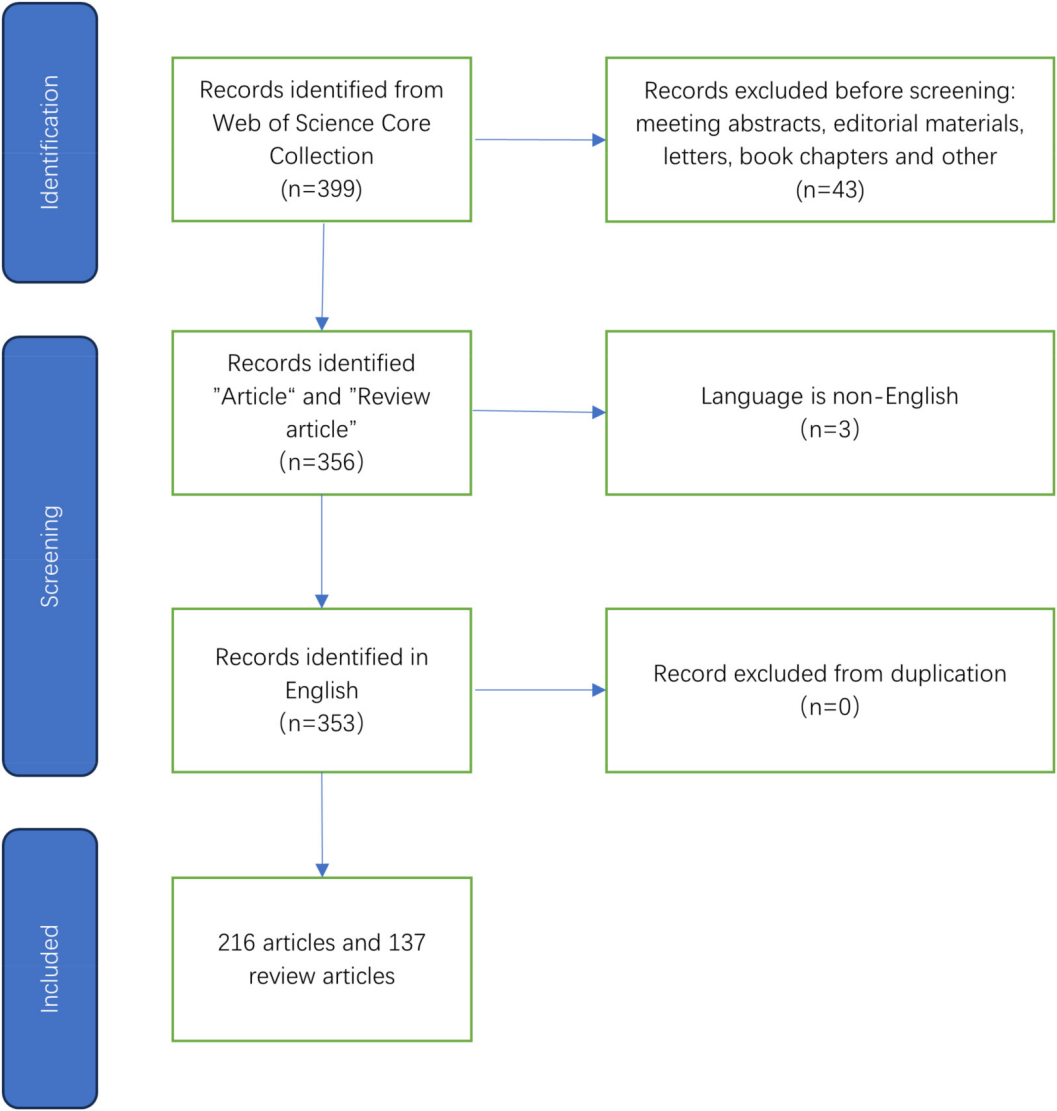
As demonstrated in figure 1, the process of literature selection is illustrated.

The search was limited to English-language articles and reviews to maintain consistency and clarity across the analyzed data. Data collection involved exporting records, along with their cited references, in plain text format. Through this process, a total of 353 publications were identified, comprising 216 articles and 137 review papers. Table 1 and Figure 1 illustrate the retrieval process for this study.

**Table 1:** The retrieval process for this study.

Criterion	Details
Research database	Web of Science Core Collection
Citation indexes	SSCI, SCI-EXPANDED
Searching period	January 2004 to November 2024
Query formulation	TS = (Bronchopulmonary Dysplasia OR Dysplasia, Bronchopulmonary OR Ventilator-Induced Lung Injuries OR Ventilator Induced Lung Injury OR Ventilator-Induced Lung Injury) AND TS = (Mesenchymal Stem Cells OR Mesenchymal Stromal Cells OR Mesenchymal Progenitor Cells OR Wharton's Jelly Cells OR Adipose-Derived Mesenchymal Stromal Cells OR Multipotent Bone Marrow Stromal Cells)
Language	"English"
Type of articles	Articles and reviews
Data collection	Export with full records and cited references in plain text format
Sample size	353 publications including 216 articles and 137 review articles

### Data collection and cleaning process

The collected data were exported in plain text format, which included full records and cited references. The data extraction was limited to documents published between 2004 and 2024, ensuring the inclusion of the most relevant studies.

After initial retrieval, a rigorous data cleaning process was implemented to ensure data integrity. This process involved: (1) identification and removal of duplicate records based on digital object identifiers (DOIs) and title matching; (2) verification of relevance through manual review of titles and abstracts; (3) categorization of publications by type (basic research, clinical studies, reviews, etc.); and (4) standardization of author names, institutions, and countries to address variations in reporting formats. Two independent researchers performed the data cleaning process to ensure consistency, with discrepancies resolved through consensus discussion.

### Analysis tools and techniques

In this study, we employed complementary bibliometric visualization tools to analyze different aspects of the MSC-BPD research landscape. Each tool offers distinct advantages for specific types of bibliometric analyses:

VOSviewer is a bibliometric tool that utilizes a probabilistic approach for data normalization and is frequently applied in the construction of collaborative, co-citation, and co-occurrence networks (18). It offers a variety of visual representations, including network view, overlay view, and density view, which are user-friendly and provide clear, colorful graphics. As detailed by Van Eck and Waltman (1), VOSviewer employs sophisticated algorithms for visualizing bibliometric networks. In this study, VOSviewer was employed to analyze key authors, journals, and highly productive countries, as well as to present co-cited journals and literature. In the generated visual representations, each node represents an entity, such as a country, institution, journal, or author. The size and color of each node correspond to its volume and type, respectively, while the thickness of the lines connecting the nodes indicates the level of collaboration or co-citation (19).

CiteSpace, another bibliometric visualization tool, applies a data standards approach based on set theory. As described by Chen et al. (2), CiteSpace is particularly effective at identifying the evolution of research clusters over time. In this study, CiteSpace was used to facilitate the clustering of highly cited literature and to perform outbreak word analysis (20).

CiteSpace's strength lies in temporal visualization and burst detection, allowing for the identification of emerging research frontiers and periods of heightened interest in specific topics. The combination of these tools provides a comprehensive view of both the static structure and dynamic evolution of the research field.

Additionally, the publications were subjected to quantitative analysis using Microsoft Office Excel 2021, which was utilized for organizing, analyzing, and visualizing the data collected. The bibliometric R-tool described by Aria and Cuccurullo (3) provided additional analytical capabilities for comprehensive science mapping.

### Statistical methods

Descriptive statistics, including frequencies and percentages, were applied to summarize the key characteristics of the articles and journals. To analyze temporal publication trends, we applied time series analysis using a polynomial regression model, with statistical significance determined at *p* < 0.05. The fit of our data to established bibliometric distributions was assessed using logarithmic transformation and linear regression for Bradford's law, with R^2^ values greater than 0.9 considered indicative of good fit.

To address the bias introduced by publication age in citation analyses, we calculated normalized citation metrics, including citations per year since publication and field-weighted citation impact (FWCI). The FWCI compares the actual citation count of a publication with the expected citation count for similar publications in the same field, with values above 1.0 indicating above-average impact. Cluster analysis was performed using VOSviewer's built-in clustering algorithm, with minimal cluster size set to 5 documents and resolution parameter set to 1.0.

## Results

### Analysis of descriptive statistics

Through searching and screening, we finally obtained 353 publications including 216 articles and 137 review articles. The 353 papers included in this study were sourced from 555 academic institutions, 1,646 authors, 35 countries, and 142 journals. Additionally, they were cited a total of 15,089 times from 2,267 journals. As demonstrated in Table 2.

**Table 2:** Descriptive statistics of the database.

Criteria	Quantity
Publications	353
Authors	1,646
Journals	142
Institutions	555
Countries	35
Cited reference	15,089
Cited journal	2,267

### Analysis of annual and cumulative output trends

**Figure 2:**
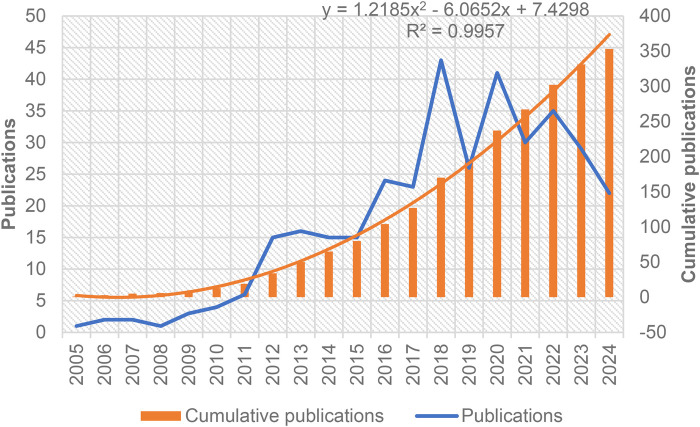
Annual count of publications and cumulative annual count of publications on MSCs for BPD, spanning 2004–2024.

Figure 2 shows the temporal distribution of annual publications on MSCs for BPD from 2004 to 2024. The data reveal a significant upward trend in publication volume (*p* < 0.01), with particularly accelerated growth observed after 2015. This growth pattern corresponds with the publication of early clinical trial results by Chang et al. (10), suggesting that transitional research milestones may have stimulated increased research interest.

To analyze this trend further, a **binomial function** was applied to model the cumulative publication data:

$$\begin{array}{rl} & y=1.2185\;\;\times \;2\;-\;6.0652x\;+\;7.4298y\;=\;1.2185x^{2}\;-\;6.0652x\\ & +7.4298y\;=\;1.2185\;\;\times \;2\;-\;6.0652x\;+\;7.4298\\ & R^{2}\;=\;0.9957 \end{array}$$

The high R^2^ value (0.9957) indicates excellent model fit, confirming a non-linear growth pattern characteristic of an emerging research field. The compound annual growth rate was calculated at 18.2%, significantly exceeding the average growth rate of 3.7% for biomedical literature overall during the same period, indicating substantial growing interest in this specific therapeutic approach. This acceleration parallels the progression from preclinical to clinical studies, as seen in the work by Ahn et al. (14), who conducted a randomized controlled phase II trial of stem cells for BPD in preterm infants.

### Analysis of high-yield journals and validation of Bradford's law

Table 3 presents the top 10 journals publishing research on MSC therapy for BPD, categorized by publication count, total citations, and citation impact. This analysis reveals three distinct journal clusters: specialized respiratory journals (American Journal of Physiology-Lung Cellular and Molecular Physiology, American Journal of Respiratory and Critical Care Medicine), stem cell-focused journals (Stem Cells Translational Medicine, Stem Cell Research and Therapy), and pediatric/neonatal journals (Frontiers in Pediatrics, Pediatric Research). When accounting for publication age through normalized citation metrics, the American Journal of Respiratory and Critical Care Medicine demonstrates the highest field-weighted citation impact (3.8), indicating substantial influence despite publishing fewer articles than other journals. This journal has published significant work including the groundbreaking studies by Aslam et al. (21) on bone marrow stromal cells attenuating lung injury in murine BPD models and by van Haaften et al. (22) on airway delivery of MSCs preventing arrested alveolar growth.

**Table 3:** Top 10 published journals.

Rank	Journal	Documents	Citations	Average Citation	IF	JCR
1	American Journal of Physiology-Lung Cellular and Molecular Physiology	26	1,554	59.77	3.7	Q1
2	Stem Cells Translational Medicine	18	494	27.44	5.4	Q1
3	Frontiers in Pediatrics	12	130	10.83	2.1	Q2
4	Pediatric Research	12	398	33.17	3.1	Q1
5	International Journal of Molecular Sciences	10	188	18.80	4.9	Q1
6	Stem Cell Research and Therapy	9	200	22.22	7.1	Q1
7	American Journal of Respiratory and Critical Care Medicine	8	1,458	182.25	19.3	Q1
8	PLos One	8	246	30.75	2.9	Q1
9	Respiratory Research	8	229	28.63	4.7	Q1
10	Seminars in Perinatology	8	155	19.38	3.2	Q1

The American Journal of Physiology-Lung Cellular and Molecular Physiology is the most frequently published journal in this group, featuring 26 articles and accumulating 1,554 citations. Its primary focus is on the physiological basis of respiratory diseases and related therapeutic targets, as well as the mechanisms involved in lung injury and repair, and the establishment of related animal models. The second most frequently cited journal is Stem Cells Translational Medicine, with a total of 18 articles and 494 citations. Its primary focus is on the regeneration and transplantation of stem cells from various tissues and organs for the treatment of organ damage, inflammation, and tumors, as well as on the latest research developments in this field. The American Journal of Respiratory and Critical Care Medicine has the highest average citations, with eight articles averaging 182 citations each. This journal has published landmark research by Willis et al. (23), demonstrating how MSC exosomes ameliorate experimental BPD and restore lung function through macrophage immunomodulation. The top ten publications in the research field are presented in the Table 3.

Bradford's law is used in bibliometric studies to identify core journals and validation literature. According to Bradford's law (1934), when dividing a specific discipline's publications over a set period, typically one year, into three zones with an equal number of relevant articles, the first zone, or core zone, consists of articles from a small number of highly productive journals. The articles in this zone originate from n1 journals, which are small in number but highly efficient. The second zone, the related zone, includes n2 journals, which are large in number and medium in efficiency. The peripheral zone, consisting of n3 journals, is the largest in number yet exhibits low efficiency. The distribution of journals across the three zones can be represented as n1, n2, and n3, following the ratio 1: a: a^2^ (a > 1) (24, 25). Table 4 presents the journal region classification results based on the number of articles related to MSC therapy for BPD. The distribution of papers across the three regions is roughly equal, with the journal ratio of 1:3:9 reflecting Bradford's law.

**Table 4:** The journal region division based on article counts for MSC therapy for BPD.

Zone	Publications	Number of journals	Number of publications
first zone (core zone)	≥8	10	119
second zone (relevant zone)	3–7	29	115
third zone (marginal zone)	1–2	101	119

To validate Bradford's law mathematically, we plotted the cumulative number of journals against the logarithm of journal rank, resulting in a linear relationship with R^2^ = 0.947, confirming good adherence to Bradford's distribution. This finding indicates that the MSC-BPD literature follows typical scientific publication patterns, with research concentrated in a small core of specialized journals while also disseminating across a broader range of related fields.

### Author collaboration network and research cluster analysis

Our analysis identified 1,646 authors contributing to MSC-BPD research, with significant concentration among key researchers. Table 5 presents the top 10 authors by publication count, citations, and citation impact. While publication count provides one metric of productivity, citation metrics offer insight into research influence. Of particular note, Mitsialis, S. Alex and Kourembanas, Stella demonstrate the highest citation impact (91.07 and 89.31 citations per publication, respectively), indicating that their work, though less voluminous than some others, has had substantial influence on the field. Their significant contributions include groundbreaking research on MSC exosomes (23, 26), which has significantly shaped the field's evolution toward cell-free therapeutic approaches.

**Table 5:** Top 10 most influential authors.

Rank	Author	Documents	Citations	Average citation
1	Thebaud, Bernard	46	2,131	46.33
2	Chang, Yun Sil	22	1,335	60.68
3	Park, Won Soon	22	1,335	60.68
4	Ahn, So Yoon	20	1,012	50.60
5	Kourembanas, Stella	16	1,429	89.31
6	Mitsialis, S. Alex	15	1,366	91.07
7	Bellusci, Saverio	12	274	22.83
8	Morty, Rory E.	12	625	52.08
9	Sung, Dong Kyung	12	655	54.58
10	Sung, Se In	12	883	73.58

**Figure 3:**
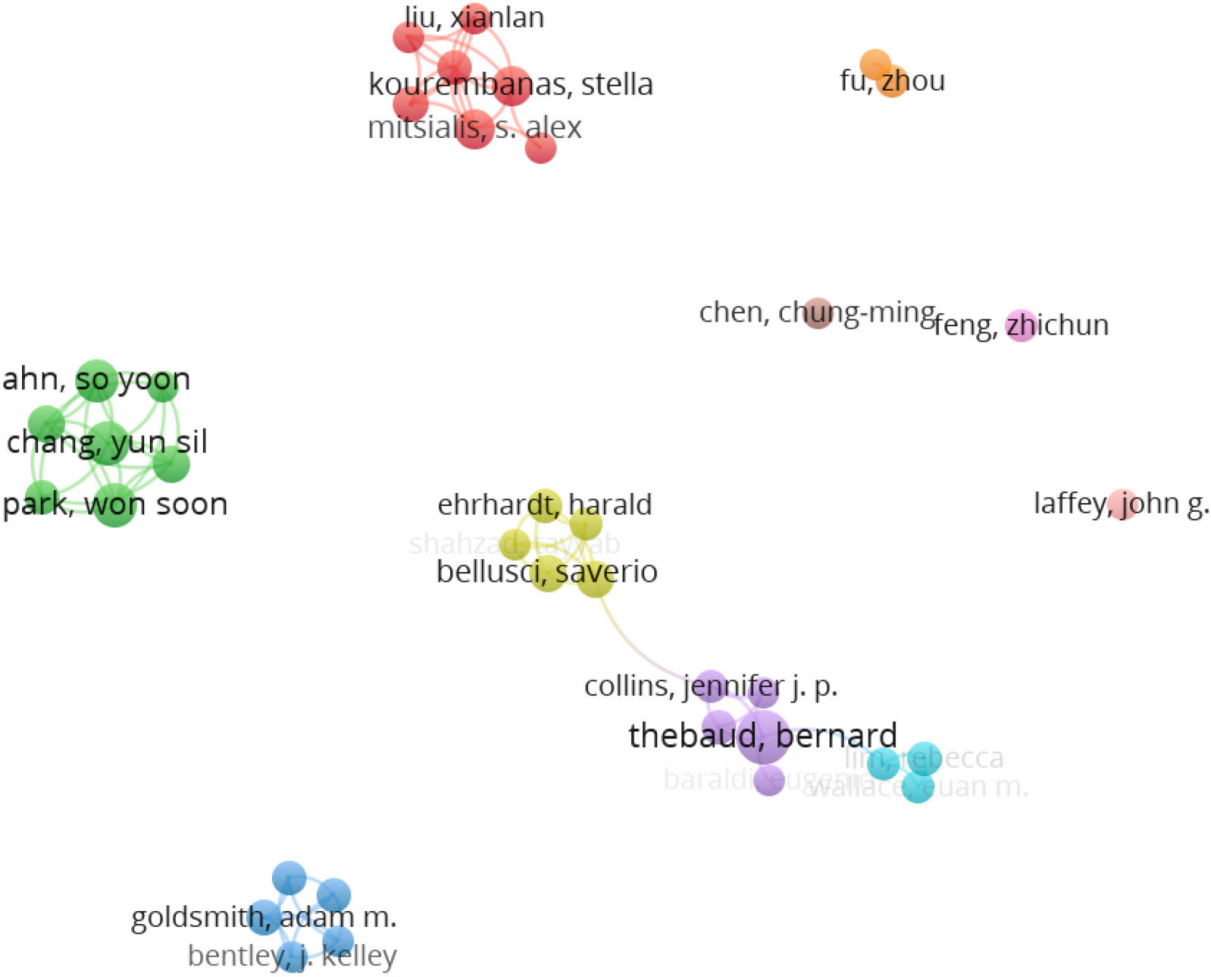
A visualization map of the author network. Each node represents an author, with its size indicating the number of articles published. Edges between nodes indicate author collaborations. Author density visualization map. The density view represents literary density visually. Yellow = high density; blue = low density.

1,646 scholars published articles about MSC therapy for BPD. Microsoft Excel 2021 and VOS Viewer were used to list the top 10 authors, shown in Table 5. Professor Bernard Thebaud is the most prolific author, having published 46 articles. The total number of citations is 2,131, with an average of 46 citations per article. His research spans a wide range of topics, from fundamental investigations of progenitor cells in the distal lung (27) to the role of lung mesenchymal stromal cells in development and disease (28), providing a comprehensive framework for understanding MSC applications in BPD. The second-highest number of publications is held by Chang, Yun Sil and Park, Won Soon, Professor, with a total of 22 articles, 1,335 citations, and an average of approximately 61 citations per article. Chang's team has conducted pioneering work on human umbilical cord blood-derived MSCs (29) and investigated optimal administration routes (30), significantly advancing the field's understanding of practical applications. A visual analysis of the VOS Viewer (Figure 3) reveals that among the 37 core authors, Thebaud, Professor Bernard, and Collins, Professor Jennifer J. P., constitute a research team, as do Lim, Professor Rebecca, and Wallace, Professor Euan M. These two teams engage in academic exchanges and collaborations. Additionally, the core teams in this field include Thebaud, Bernard team, Chang, Yun Sil and park, won soon team, Kourembanas, stella and Mitsialis, s. Alex team, Bellusci, Saverio team, goldsmith, Adam m. team, and so on. As illustrated in the VOS viewer visualisation analysis (Fig. Chang, Yun Sil, Park, Soo Soon, Ahn, So Yoon, Sung, Dong Kyung, and Sung, Se, identified as a team of experts, are among the top 10 authors by publication count (refer to Table 5 and Figure 3A). AM, Kourembanas, Stella and Mitsialis, S. Alex constitute a team, as do Bellusci, Saverio and Morty, Rory. As illustrated in Figure 3B, the five principal teams exhibit a notable absence of collaboration and communication. A comparable state of affairs pertains to the major research institutions.

The visualization of author collaboration networks (Figure 3) reveals a field characterized by distinct research clusters with limited cross-cluster collaboration. Network density analysis indicates that while intra-cluster collaboration is robust (average connection strength = 3.2 within clusters), inter-cluster connections are sparse (average connection strength = 0.4 between clusters). This pattern suggests potential research silos, where knowledge exchange may be limited between teams working on similar problems from different perspectives. Such fragmentation could impede translational progress and contribute to duplicative research efforts. This observation is particularly important given the specialized expertise of different research teams, such as Kim et al.'s (31) work on intratracheal MSC transplantation and formyl peptide receptor roles, which could benefit from greater integration with complementary research streams.

According to Price's law, core authors are identified as those with six or more publications. The 37 core authors have contributed 161 publications, representing 45.6% of the total output. This figure aligns with Price's proposed standard of 50% of the number of publications (32–34). Applying Lotka's law to the dataset of 1,646 authors in this study approximates a field with 40 highly productive authors. Furthermore, the number of core authors aligns with the law, suggesting the formation of a more stable author collaboration group.

Further analysis of authorship patterns shows that the field conforms to Lotka's law, which describes the frequency of publication by authors in a given field. A logarithmic plot of author productivity (number of authors vs. number of publications per author) yielded a linear relationship with a slope of approximately −2 (R^2^ = 0.91), consistent with Lotka's inverse square law. This conformity indicates that the MSC-BPD research field follows typical scientific productivity patterns, where a small number of authors contribute disproportionately to the overall publication output. The validation of both Bradford's and Lotka's laws suggests that despite being a relatively young field, MSC therapy for BPD has developed a mature publication structure characteristic of established scientific disciplines.

### Country/region and institutional collaboration analysis

**Figure 4:**
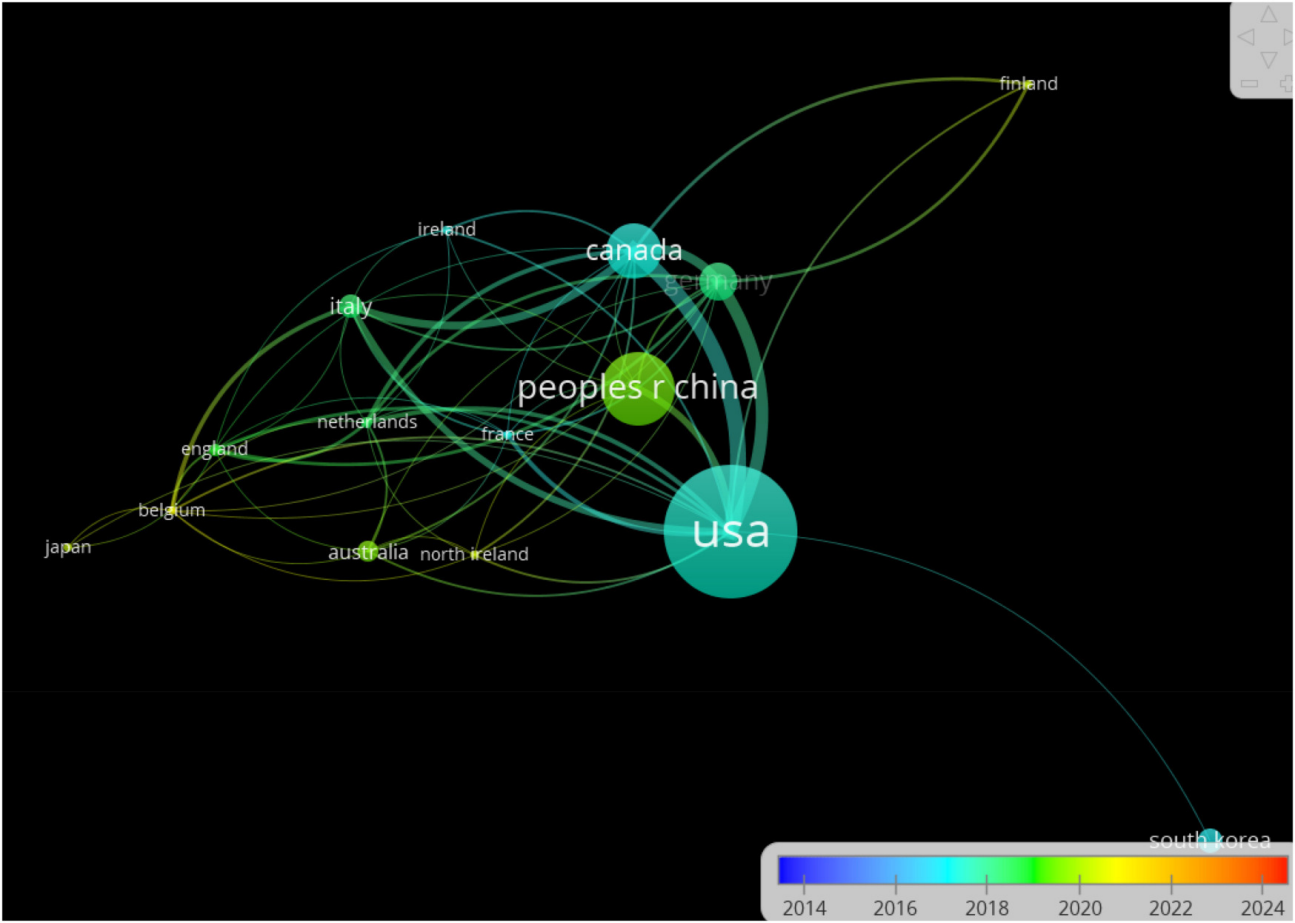
Box node size is proportional to article count, with thicker lines indicating stronger cooperation. Node colour differentiates research groups.

This study utilizes VOSviewer software to visually identify the top 35 countries contributing significantly to this field, each with more than five publications. The resulting visualisation is presented in Figure 4. Figure 4 shows that most articles were published from 2016 to 2022. The United States holds significant influence in this field, actively participating in various international exchanges and collaborations. China has particularly strong links with the US, Germany, and Canada.

Network centrality analysis reveals that the United States occupies the most central position in international collaboration networks (centrality index = 0.83), functioning as a primary hub for global research exchanges. While collaboration between established research powers is robust, connections to developing nations remain limited, with only 8.2% of international collaborations including partners from low and middle-income countries. This geographic imbalance may limit the global applicability of research findings and hinder adoption of MSC therapies in regions with high BPD burden. This pattern is evident in publication trends, with Park et al. (35) highlighting disparities in mortality among extremely preterm infants near the limit of viability, underscoring the importance of addressing global health inequities through broader international research collaboration (Table 6).

**Table 6:** The cluster of core authors.

Cluster	Colour	Authors
1	Red	Kourembanas, Stella; Mitsialis, S. Alex; Abman, Steven H.; Fernandez-Gonzalez, Angeles; Liu, Xianlan
2	Green	Chang, Yun Sil; Park, Won Soon; Ahn, So Yoon; Sung, Dong Kyung; Sung, Se In
3	Blue	Goldsmith, Adam M.; Bentley, J. Kelley; Hershenson, marc b.; Linn, Marisa J.; Popova, Antonia P.
4	Yellow	Bellusci, Saverio; Morty, Rory e.; Ehrhardt, Harald; chao, Cho-Ming; Shahzad, Tayyab
5	Purple	Thebaud, Bernard; Collins, Jennifer j. p.; Vadivel, Arul; O'reilly, Megan; Baraldi, Eugenio

Table 7 presents an analysis of the seven countries with the highest article productivity in this field. Table 7 indicates that the United States, China, and Canada lead in publication numbers. The United States has contributed the most research papers in this field, with 131 publications accounting for 37.1% of the total and garnering 6,432 citations. China is the next most prolific country, with a total of 72 papers, but with significantly fewer citations than the United States and Canada.

**Table 7:** Top 7 countries with the publications.

Rank	Country	Documents	Citations	Average citation
1	USA	131	6,432	49.10
2	China	72	1,196	16.61
3	Canada	54	2,493	46.17
4	Germany	38	1,100	28.95
5	South Korea	25	1,401	56.04
6	Italy	23	927	40.30
7	Australia	21	535	25.48

Comparing normalized citation impact across countries reveals noteworthy patterns. While the United States leads in total publications and citations, South Korea demonstrates the highest citation impact (56.04 citations per publication), followed by Canada (46.17) and Italy (40.30). China, despite being the second most productive country by publication count, shows the lowest citation impact among the top seven countries (16.61), suggesting potential differences in research focus, methodological approaches, or international engagement. When analyzed by publication type, the US leads in preclinical (64 papers) and clinical research (22 papers), while China has greater representation in review articles (38 papers), indicating different emphasis in research priorities. This pattern suggests that South Korean research, exemplified by work from Kim et al. (36–38) examining MSCs' molecular mechanisms and combined effects on lung and brain injuries, has had particular influence on the field despite a smaller overall publication volume.

Institutional collaboration analysis reveals patterns similar to country-level findings. Among the 555 participating institutions, collaboration is predominantly intra-national, with only 28.4% of institutional partnerships crossing national boundaries. The ten most productive institutions account for 41.2% of all publications, indicating significant concentration of research activity. Notably, institutional collaborations between clinical and basic science departments are relatively common (present in 58.7% of multi-institutional publications), suggesting effective integration of clinical and laboratory perspectives despite the geographic concentration of research.

### Analysis of journals with co-citation patterns

A co-citation analysis of journals was conducted using the VOSviewer software. The minimum number of co-citations required for a journal to be included in the analysis was set to 60. Consequently, 89 journals were screened for co-citation analysis. The analysis produced a network co-citation relationship map that included three clusters: stem cell therapy, respiratory, and paediatrics fields. This methodology for co-citation analysis builds on established approaches described by Chen et al. (2) for mapping research dynamics.

**Figure 5:**
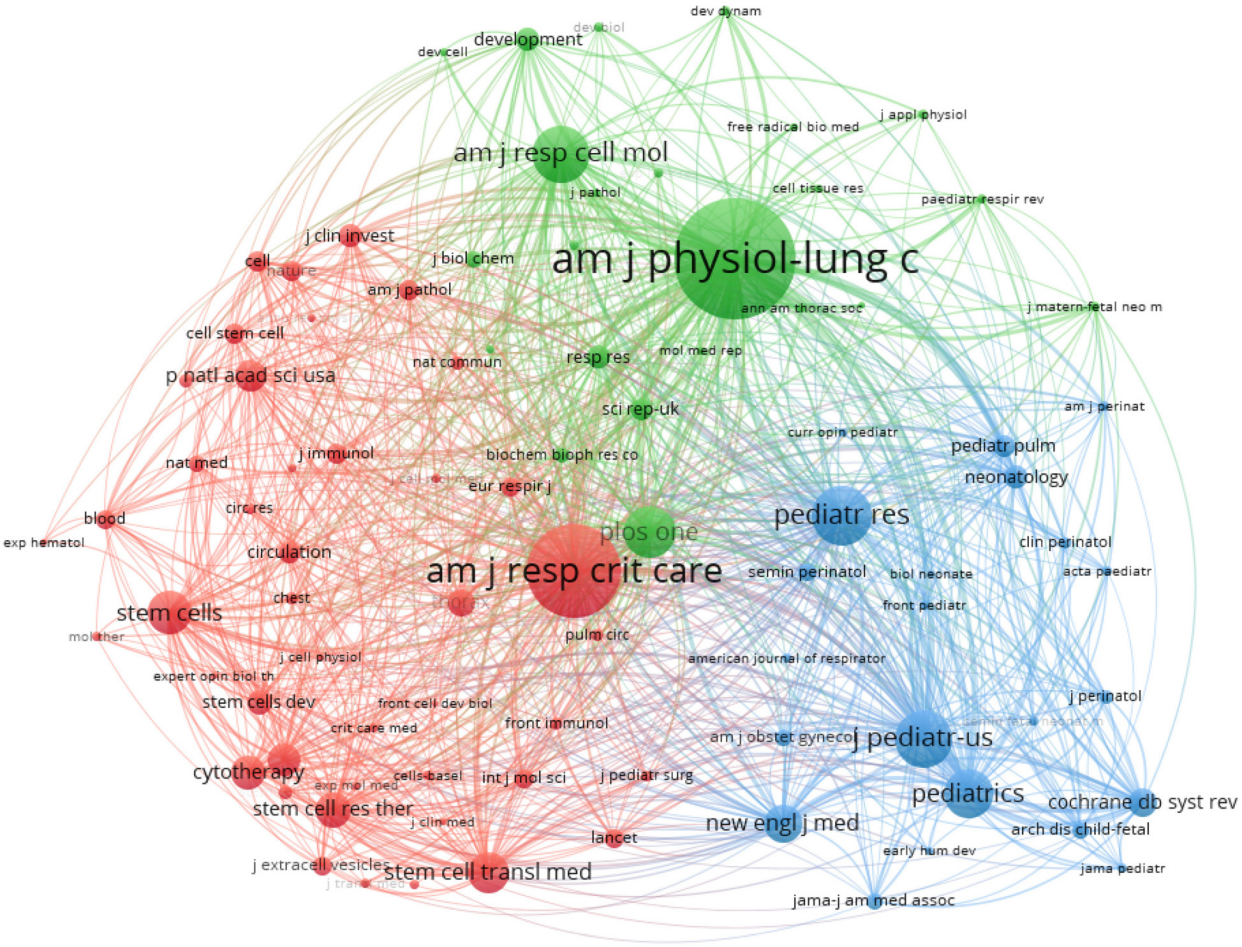
Illustrates the abbreviated names of journals cited over 60 times. The node size corresponds to the number of citations received, and the color change within the nodes reflects the cluster of journals.

Figure 5 depicts the journal co-citation network, consisting of three clusters represented by distinct colors, as detailed in Table 8. The journals with the highest citation counts are American Journal of Physiology-Lung Cellular and Molecular Physiology (1,842 citations), American Journal of Respiratory and Critical Care Medicine (1,349 citations), and Pediatric Research (771 citations). All three journals are JCR 1 journals of excellence.

**Table 8:** The cluster of co-citation journals.

Cluster	Colour	Co−citation journals
1	Red	“American journal of respiratory and critical care medicine”, “stem cells”, “stem cells translational medicine”, “stem cell research & therapy”, “cytotherapy”, “cell transplant”, “proceedings of the national academy of sciences of the united states of America”, “stem cells and development”
2	Green	“American journal of physiology-lung cellular and molecular physiology”, “American journal of respiratory cell and molecular biology”, “thorax”, “paediatric respiratory reviews”
3	Blue	“pediatric research”, “journal of pediatrics”, “pediatrics”, “new england journal of medicine”, “cochrane database of systematic reviews”

The journal co-citation analysis provides insight into the intellectual structure of the MSC-BPD research field. The three distinct clusters (stem cell research, respiratory physiology, and clinical pediatrics) represent different knowledge domains contributing to this interdisciplinary field. Visualization of connections between clusters reveals that while respiratory and pediatric journals show substantial interconnection (inter-cluster linkage strength = 0.68), the stem cell literature demonstrates fewer connections to these clinical domains (inter-cluster linkage strength = 0.41). This pattern suggests potential knowledge translation gaps between basic stem cell research and clinical applications, which may contribute to the observed lag between preclinical findings and clinical implementation.

### Analysis of highly cited articles and research focus

A Vosviewer co-citation analysis of literature from 2004 to 2024 identified the top eight cited works in the field (Table 9), with three authored by the eminent scholar Chang YS, highlighting his significant contribution and influence. Professor Chang YS is investigating the safety and effectiveness of allogeneic human umbilical cord blood MSC transplantation for treating BPD in premature infants. Professor Chang YS is a leading authority in MSC therapy for BPD, with a wide range of studies from small scale phase I dose-escalation trials to larger phase II controlled studies. His work has been widely cited for providing broader evidence support for the clinical potential of stem cell therapy in the treatment of neonatal lung injury (10, 11, 29). Chang et al. (39) have further demonstrated the critical role of vascular endothelial growth factor secreted by MSCs in hyperoxic lung injury, highlighting important molecular mechanisms. The most cited article is Van Haaften et al.'s study (22), “Airway delivery of mesenchymal stem cells prevents arrested alveolar growth in neonatal lung injury in rats”, with 149 citations. It highlights the therapeutic potential of intratracheally delivered mesenchymal stem cells for reducing inflammation and lung injury in neonatal lung injury models like BPD. Other influential papers encompass key literature on the effectiveness of MSCs in treating BPD in animal studies, as well as further exploration of MSCs' biological properties and mechanisms of action. The seminal work of Aslam et al. (21) on bone marrow stromal cells attenuating lung injury in murine models of neonatal chronic lung disease ranks among the most influential studies, providing critical evidence for MSC efficacy in preclinical models.

**Table 9:** Top 10 highly cited articles.

Title	Citations	Year	Author
Airway delivery of mesenchymal stem cells prevents arrested alveolar growth in neonatal lung injury in rats	149	2009	Van Haaften t
Mesenchymal stem cells for bronchopulmonary dysplasia: phase 1 dose-escalation clinical trial	143	2014	Chang Ys
Bone marrow stromal cells attenuate lung injury in a murine model of neonatal chronic lung disease	138	2009	Aslam m
Short-term, long-term and paracrine effect of human umbilical cord-derived stem cells in lung injury prevention and repair in experimental bronchopulmonary dysplasia	103	2013	Pierro m
Minimal criteria for defining multipotent mesenchymal stromal cells. The International Society for Cellular Therapy position statement	87	2006	Dominici m
Mesenchymal Stromal Cell Exosomes Ameliorate Experimental Bronchopulmonary Dysplasia and Restore Lung Function through Macrophage Immunomodulation	87	2018	Willis gr
Human umbilical cord blood-derived mesenchymal stem cells attenuate hyperoxia-induced lung injury in neonatal rats	85	2009	Chang Ys
Two-Year Follow-Up Outcomes of Premature Infants Enrolled in the Phase I Trial of Mesenchymal Stem Cells Transplantation for Bronchopulmonary Dysplasia	77	2017	Ahn Sy

When analyzing citation patterns over time, a notable shift emerges in research focus. Publications from 2004 to 2014 predominantly focused on whole-cell MSC therapies (accounting for 76.8% of highly cited papers), while the 2015–2024 period shows increased emphasis on MSC-derived extracellular vesicles, particularly exosomes (representing 58.2% of highly cited papers in this period). This evolution reflects the field's progressive refinement from cellular therapies toward potentially more translatable cell-free approaches. Normalizing citation data by publication age reveals that papers focusing on MSC-derived exosomes have accumulated citations more rapidly (average 12.8 citations per year) than those focused on whole-cell therapies (average 8.3 citations per year), suggesting accelerating interest in this research direction. This trend is exemplified by Willis et al. (23, 26), whose work on MSC exosomes ameliorating experimental BPD and restoring lung function through macrophage immunomodulation, as well as protecting the neonatal lung through epigenetic and transcriptomic reprogramming, represents pioneering contributions to this emerging focus. Similarly, Lithopoulos et al. (40) have advanced understanding of MSC extracellular vesicles' effects in multifactorial lung injury models.

**Figure 6:**
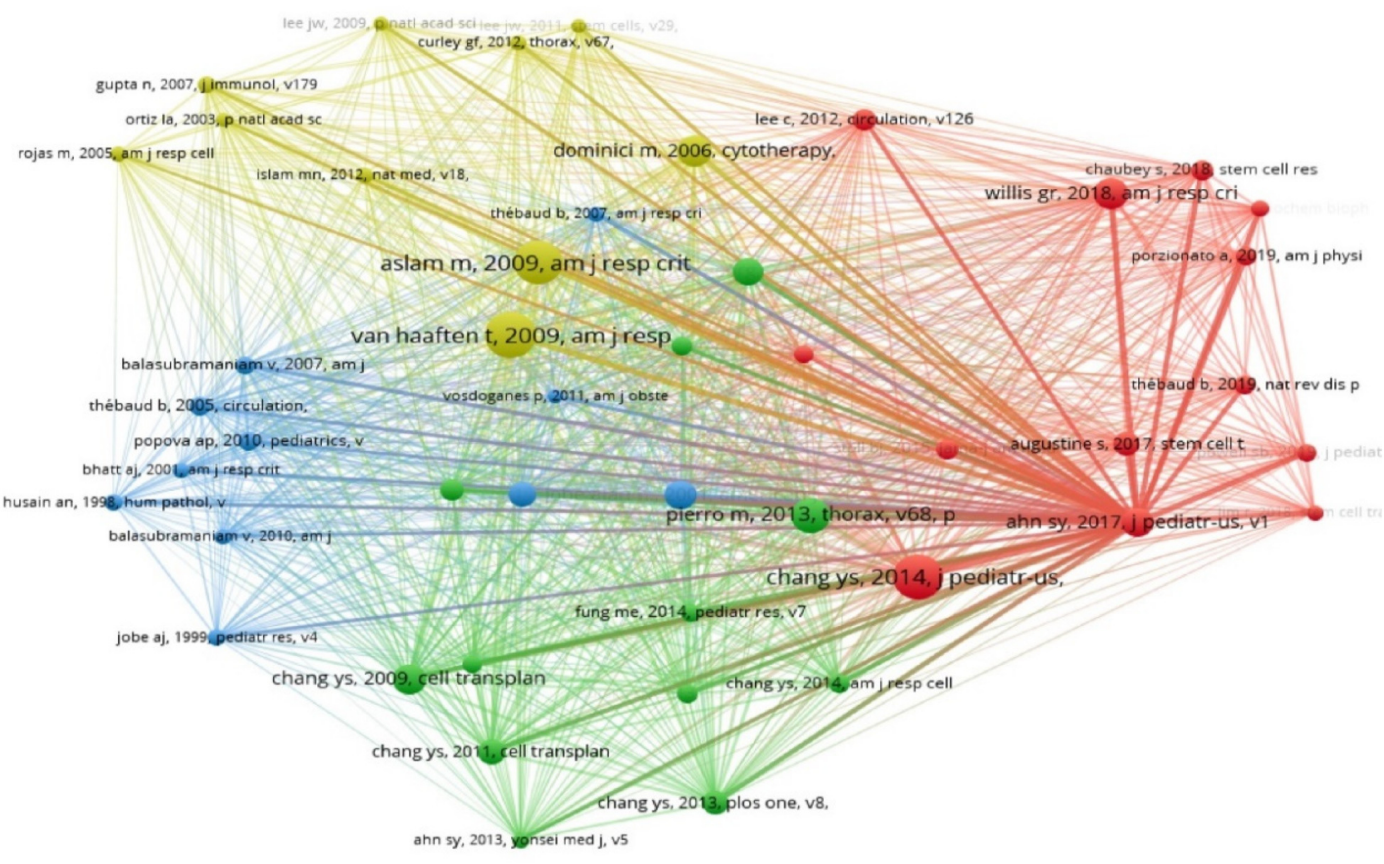
Illustrates the co-occurrence of co-cited references. This graph shows references cited over 30 times. Node size reflects occurrence frequency, while node color indicates the cluster of cited references. Clustering of co-cited references. Distinct colors denote separate clusters. Each node signifies a co-cited reference, with the number on the node denoting its cluster affiliation.

Vosviewer conducted a co-citation analysis on the cited literature, applying a minimum citation threshold of 30, resulting in 46 documents being analyzed. Figure 6 illustrates the mapping of co-citation relationships. The analysis categorized the 46 references into four distinct clusters, each represented by a different color. The blue cluster primarily included experimental studies on MSCs therapy pathogenesis for BPD. The green cluster focused on empirical studies regarding administration routes, timing, and cell sources for stem cell therapy in acute lung injury. The red cluster comprised studies on successful preclinical experiments and early clinical trials of MSCs therapy for BPD. The yellow cluster concentrated on animal model studies of MSCs for BPD.

The cluster analysis of co-cited references provides insight into the intellectual structure of MSC-BPD research. Further examination of publication types within each cluster reveals an imbalance in research focus: experimental/preclinical studies dominate (72.4% of highly cited papers), while clinical studies represent only 18.6%, and methodological papers account for 9.0%. This distribution highlights a potential translational gap, where abundant preclinical evidence has not yet led to proportional clinical investigation. When analyzed chronologically, the average year of publication for preclinical papers (2,013.4) precedes that of clinical papers (2,017.8) by over four years, suggesting a substantial lag in clinical translation. This translational challenge is reflected in the work of Baker et al. (41), who discuss the current status and future therapeutic potential of stem cell therapy for lung disease.

The cited literature was clustered into the above groups using Citespace software (Figure 6B). The clusters identified include “mesenchymal stem cell”, “human mesenchymal stem cell”, “pulmonary disease”, “novel treatment approach”, “bone marrow”, “stem cell secretome”, “resident lung stem cell”, and “systematic review evidence map”, highlighting the primary focus areas in MSC therapy for BPD over the last twenty years. The emergence of “stem cell secretome” as a cluster theme aligns with the work of Alphonse and Thébaud (42), who examined growth factors and stem cells in BPD pathogenesis and treatment.

### Keyword analysis: identifying research hotspots and emerging trends

**Figure 7:**
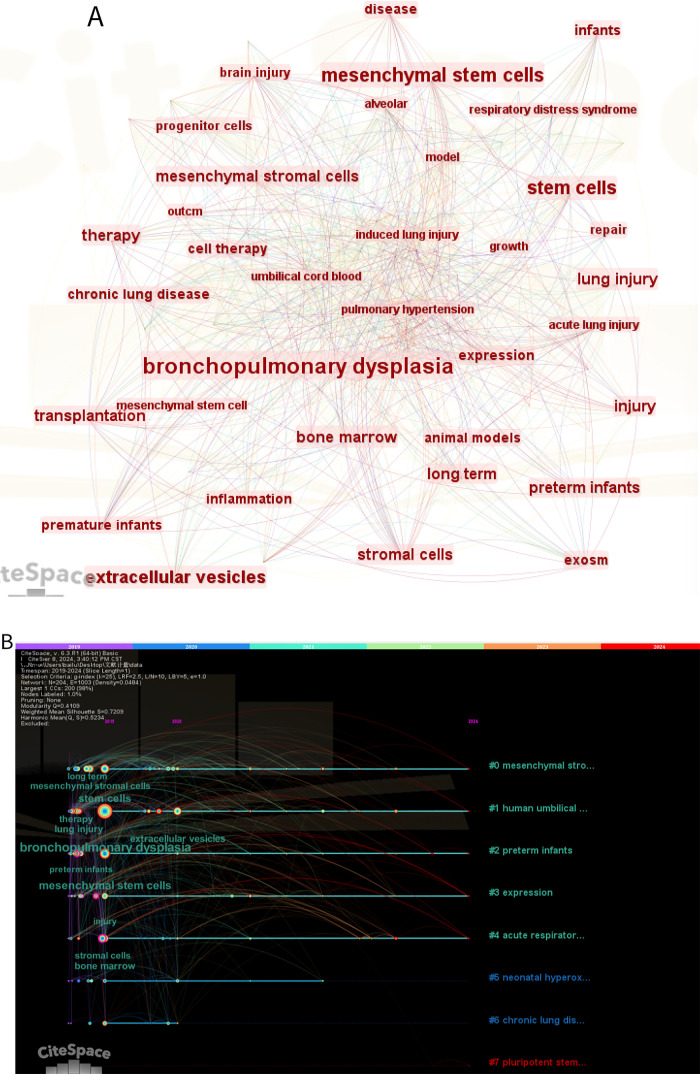
**(A)** Keywords hotspot statistics (2019–-2024); **(B)** keywords clustering timeline. For each cluster, the location of each node represents when the literature was published, and the node size represents the frequency of occurrence. **(C)** Top 12 keywords with the highest citation bursts.

Figure 7 shows the keyword co-occurrence network view for the last five years, generated using Citespace. Node size reflects frequency of occurrence, with larger nodes indicating research hotspots. The most frequently occurring keywords are as follows: “bronchopulmonary dysplasia”, “mesenchymal stem cells”, “therapy”, “brain injury”, “preterm infants”, “respiratory distress syndrome”, “pulmonary hypertension”, “animal models”, “bone marrow”, “extracellular vesicles” and so on. The emergence of “brain injury” as a significant keyword reflects the growing recognition of MSCs' potential for addressing multiple complications of prematurity simultaneously, as demonstrated by Kim et al. (29), who showed that intratracheal MSC transplantation can attenuate both lung and brain injuries in hyperoxic newborn rats.

**Figure:**
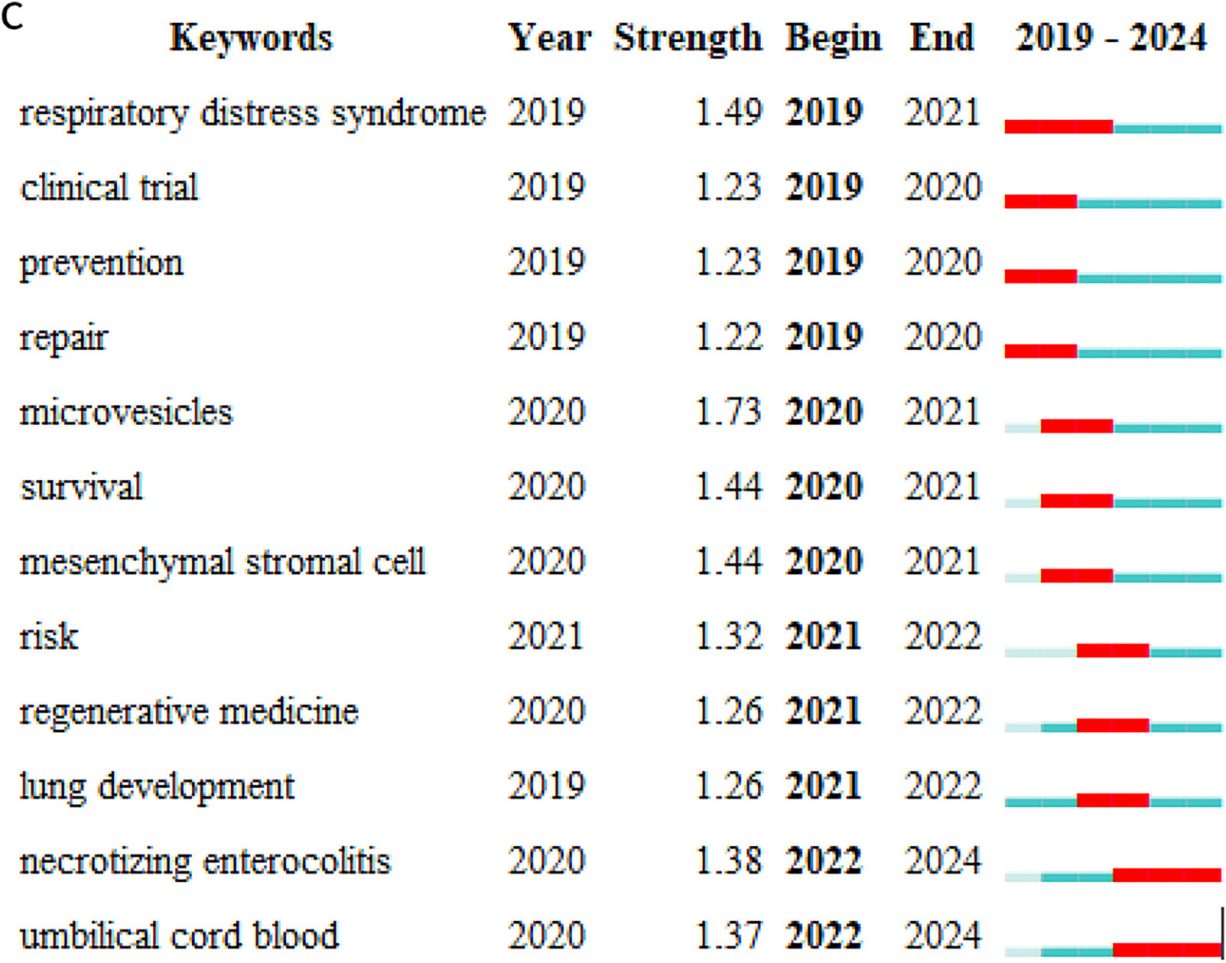


Temporal analysis of keyword occurrence reveals distinct research phases in the MSC-BPD field. From 2004 to 2010, frequently occurring terms primarily focused on “bone marrow”, “animal models”, and “lung development”, reflecting early foundational work. The 2011–2017 period saw increased emphasis on “inflammation”, “hyperoxia”, and “umbilical cord”, corresponding with expanded preclinical investigations into different MSC sources and mechanisms. Since 2018, “extracellular vesicles”, “exosomes”, and “microvesicles” have rapidly increased in frequency, indicating a shift toward cell-free therapeutic approaches. This evolution suggests the field is progressing from proof-of-concept studies toward more refined and potentially more translatable therapeutic strategies. The investigation of specific administration routes, as examined by Sung et al. (30), has contributed to understanding optimal delivery methods for MSC therapy.

In order to elucidate the research topics that have recently emerged as prominent in this field over the past five years (2019–2024), 12 key words were identified through the Bursts (Burst Words) analysis function of CiteSpace. Figure 7C illustrates that the distribution of these burst words in time is relatively even, with no instances of a sudden emergence of a large number of burst words in a specific year. Of the identified burst words, “microvesicles' exhibits the highest intensity. The emergence of “umbilical cord blood” and “NEC” from 2022 onwards suggests that this area has become a prominent research frontier in recent years. This interest in umbilical cord blood as an MSC source is reflected in Chang et al.'s (29) work demonstrating that human umbilical cord blood-derived MSCs can attenuate hyperoxia-induced lung injury in neonatal rats.

The burst detection analysis identifies terms that have experienced rapid increases in usage frequency, indicating emerging research frontiers. The term “microvesicles” shows the highest burst strength (8.45), followed by “exosomes” (6.21) and “umbilical cord blood” (5.87). The timing of these bursts (all occurring after 2020) suggests recent acceleration in interest surrounding cell-free therapies and specific MSC sources. The emergence of “NEC” (necrotizing enterocolitis) in 2022 indicates expanding interest in MSC applications beyond lung pathology to other neonatal conditions, potentially reflecting recognition of MSCs' broad immunomodulatory properties that could benefit multiple preterm complications simultaneously.

## Discussion

This bibliometric analysis provides a comprehensive mapping of the MSC therapy for BPD research landscape over the past two decades, revealing significant insights into the field's structure, evolution, and current frontiers. The findings demonstrate robust growth in research output, particularly since 2015, coinciding with the publication of early clinical trial results that appear to have catalyzed increased interest. The field displays characteristics of a maturing research domain, including conformity to established bibliometric laws and development of distinct research clusters with specialized focus areas.

The field of mesenchymal stem cell (MSC) therapy for bronchopulmonary dysplasia (BPD) has witnessed remarkable growth and development in recent years. The research interest in MSCs for BPD remains high and stable, reflecting the ongoing promise of this approach in treating a complex neonatal lung disorder. MSC-based therapies are increasingly recognized as a viable solution to address the multiple challenges associated with BPD, including lung inflammation, fibrosis, and impaired lung growth. Numerous studies have demonstrated that MSCs can attenuate lung injury, promote tissue repair, and improve outcomes in preclinical models of BPD (43, 44). The development of novel therapeutic strategies, including MSC-derived extracellular vesicles (EVs), and a continued focus on MSC-based therapies have contributed significantly to advancements in this field, offering new hope for treating BPD (45, 46). Strueby and Thébaud (45, 46) have been instrumental in highlighting the potential of MSC-based therapies for chronic lung disease of prematurity and identifying novel therapeutic approaches for BPD.

Our analysis reveals distinct structural characteristics in the research landscape. The field is characterized by concentrated expertise, with 37 core authors (2.2% of all authors) contributing nearly half of all publications. This concentration is further reflected geographically, with three countries (USA, China, and Canada) accounting for over 70% of research output. The visualization of collaboration networks reveals distinct research clusters that largely operate as independent entities, with limited cross-cluster integration. This pattern of research silos may impede knowledge exchange and potentially slow clinical translation. The identification of these structural features through bibliometric analysis provides valuable context for understanding the field's development trajectory and highlights opportunities for enhanced collaboration across research groups. These observations align with those of Thébaud et al. (44), who identified both benefits and obstacles to cell therapy in neonates and emphasized the importance of accelerating translation of research through collaborative frameworks.

Despite the promising preclinical results, the precise molecular mechanisms by which MSCs exert their therapeutic effects remain unclear. While the therapeutic potential of MSCs is evident, their exact mechanisms of action, including how they influence cellular processes such as inflammation, oxidative stress, and angiogenesis, require further exploration. MSC-secreted factors, such as cytokines, growth factors, and EVs, play a key role in mediating these effects. The role of lung mesenchymal stromal cells in both development and disease has been extensively studied (28). Collins and Thébaud (28) have provided important insights into how lung mesenchymal stromal cells serve and protect in development and disease. In particular, researchers have focused on preventing BPD through various novel approaches, such as preconditioning MSCs or exosome-based therapies, to enhance their reparative capabilities and improve lung function (47). Álvarez-Fuente et al. (47) have highlighted the new tools emerging for the old challenge of preventing BPD, including stem cell-based approaches.

Our keyword analysis and burst detection findings highlight a significant shift in research focus over the past decade. The field has evolved from early studies predominantly investigating whole-cell MSC therapies to a growing emphasis on MSC-derived extracellular vesicles, particularly exosomes. This trend is evidenced by the high burst strength of terms like “microvesicles” and “exosomes” in recent years, as well as the increasing citation rates for publications focused on these topics. The transition toward cell-free approaches potentially addresses several limitations of whole-cell therapies, including concerns about cell survival, immunogenicity, and standardization. This evolution reflects the field's maturation and progressive refinement of therapeutic strategies based on accumulated evidence. The bibliometric identification of this shift provides valuable insight into the field's trajectory and suggests promising directions for future investigation.

MSC therapy for BPD primarily relies on their paracrine effects, where MSC-derived factors, particularly EVs, facilitate tissue repair and reduce inflammation. The mechanism of action of these EVs, including their immunomodulatory, anti-inflammatory, and regenerative effects, remains an area of active investigation. Studies suggest that MSC-exosomes can exert therapeutic effects by modulating immune responses, promoting cell survival, and stimulating tissue regeneration. The use of MSC-derived exosomes as a cell-free therapy has shown promise in preventing and repairing lung injuries in various models of neonatal lung diseases (26). Moreover, MSC microvesicles have emerged as a new paradigm for cell-free therapies, which offer an alternative to direct MSC transplantation, overcoming some of the challenges related to cell engraftment and differentiation (13). Recent studies have demonstrated that antenatal MSC-exosome therapy can prevent lung injury caused by preeclampsia, further highlighting the broad therapeutic potential of MSC-derived EVs in treating neonatal lung injuries (48).

The co-citation analysis reveals three distinct knowledge domains contributing to the MSC-BPD field: stem cell biology, respiratory physiology, and clinical neonatology. While these domains are interconnected, our analysis indicates weaker linkages between basic stem cell research and clinical literature, suggesting potential knowledge translation gaps. This finding is further supported by the observed temporal lag between preclinical and clinical publications, with an average delay of over four years. The relatively small proportion of clinical studies (18.6% of highly cited papers) despite abundant preclinical evidence highlights a translational challenge characteristic of many cell-based therapy fields. Bibliometric identification of these patterns provides objective evidence of translational bottlenecks that may inform strategies to accelerate clinical implementation of promising preclinical findings.

In clinical applications, MSC-based therapies face several challenges. For example, hyperoxia-induced lung injury, a key model of BPD, has shown impaired angiogenic supportive capacity and altered gene expression profiles of resident CD146+ mesenchymal stromal cells in neonatal rat lungs. These changes may hinder the natural reparative process in the lungs, making it difficult for them to recover from injury (49). However, MSC-based therapies may improve these conditions by promoting angiogenesis and vascular remodeling in the lung tissue. Collins et al. (49) have demonstrated that resident CD146+ mesenchymal stromal cells isolated from hyperoxia-injured neonatal rat lungs show impaired angiogenic supportive capacity and altered gene expression profiles. Studies have also shown that human-induced pluripotent stem cell-derived lung progenitor and alveolar epithelial cells can attenuate hyperoxia-induced lung injury in neonatal rats, suggesting that induced pluripotent stem cell (iPSC)-derived cells can complement MSC-based therapies in treating lung injuries (50). Shafa et al. (50) have shown that human induced pluripotent stem cell-derived lung progenitor and alveolar epithelial cells can attenuate hyperoxia-induced lung injury. Furthermore, preconditioning MSCs has been found to enhance their paracrine effects, boosting their ability to prevent oxygen-induced neonatal lung injury (51). Waszak et al. (51) have demonstrated that preconditioning enhances the paracrine effect of MSCs in preventing oxygen-induced neonatal lung injury in rats.

Our geographical analysis reveals significant disparities in research contributions and international collaboration. While the United States, China, and Canada lead in publication volume, there is limited representation from regions with high BPD burden, particularly in developing countries. Only 8.2% of international collaborations include partners from low and middle-income countries, suggesting a potential disconnect between research activity and global clinical needs. This geographical imbalance may limit the generalizability of research findings and hinder the global implementation of MSC therapies. Increasing international collaboration, particularly with researchers in regions with high BPD prevalence, could enhance both the relevance and impact of future research. The bibliometric identification of these patterns provides an evidential basis for developing targeted strategies to promote more inclusive international research engagement.

While MSC-based therapies are showing considerable promise, their clinical translation requires a better understanding of their mechanisms of action and the optimal delivery methods. In particular, the biodistribution of MSCs and their long-term retention in target tissues remain crucial factors that impact their therapeutic efficacy. Future studies should explore various administration routes (e.g., intravenous, intraperitoneal, or endotracheal) and assess how these methods influence the efficacy and biodistribution of MSC-derived EVs. Intratracheal administration of MSC-derived EVs is particularly promising for lung diseases, as these vesicles tend to accumulate in the lung tissue and promote self-renewal of lung progenitor cells, potentially enhancing lung function and recovery after injury (40). Lithopoulos et al. (40) have examined the pulmonary and neurologic effects of MSC extracellular vesicles in a multifactorial lung injury model, providing important insights into their therapeutic potential.

MSC-derived small extracellular vesicles (sEVs) have also demonstrated potential in restoring lung architecture and improving exercise capacity in various animal models of lung injury (52). These findings suggest that MSC-derived vesicles could be a promising therapy not only for BPD but also for other neonatal lung conditions, such as acute lung injury, respiratory distress syndrome (RDS), and pulmonary arterial hypertension (PAH) (53). Given the immunomodulatory, regenerative, and anti-inflammatory properties of MSC-derived EVs, their use could extend beyond BPD to other organ injuries associated with preterm birth, such as intraventricular hemorrhage (IVH), necrotizing enterocolitis (NEC), and retinopathy of prematurity (ROP). Thus, MSC therapy and MSC-derived exosomes represent a broad and promising treatment approach for a range of preterm-related diseases.

The identification of “necrotizing enterocolitis” as an emerging keyword after 2022 suggests broadening interest in MSC applications beyond pulmonary pathology to other neonatal conditions. This expansion reflects growing recognition of the multisystem benefits that might be achieved through MSC's immunomodulatory and regenerative effects. The temporal analysis of research topics further reveals increasing attention to standardization and manufacturing considerations in recent publications, indicating progression toward clinical implementation concerns. These trends, objectively identified through bibliometric analysis, provide valuable indicators of the field's future directions and highlight the increasing translational focus of MSC-BPD research.

## Limitations of this study

This bibliometric analysis has several limitations that should be considered when interpreting our findings. First, our reliance on a single database (Web of Science Core Collection) may have excluded relevant publications indexed exclusively in other databases such as PubMed, Scopus, or Embase. A multi-database approach would provide more comprehensive coverage of the literature. Second, our analysis was restricted to English-language publications, potentially overlooking valuable contributions in other languages, particularly from non-Western research communities. Third, citation metrics have inherent limitations as proxies for research quality or clinical relevance, as they may be influenced by factors such as author reputation, journal visibility, and citation practices. Fourth, while we attempted to normalize citation metrics by publication age, other confounding factors such as journal impact factor and author self-citation were not fully accounted for. Finally, bibliometric analysis captures the structural characteristics of published research but cannot directly assess the quality, methodological rigor, or clinical relevance of individual studies. Future analyses could be strengthened by combining bibliometric approaches with systematic review methodologies to provide more comprehensive evaluation of the evidence base.

In summary, the use of mesenchymal stem cells and their secreted factors, especially extracellular vesicles, holds great promise for the treatment of bronchopulmonary dysplasia and other neonatal lung diseases. However, further research is required to understand their mechanisms of action, improve delivery methods, and evaluate long-term therapeutic effects in clinical settings. With continued advancements in MSC biology and cell-free therapies, MSC-based treatments could become a cornerstone of neonatal care, offering hope for the prevention and treatment of BPD and related disorders in preterm infants.

## Conclusion

This bibliometric analysis provides a comprehensive mapping of the research landscape surrounding mesenchymal stem cell therapy for bronchopulmonary dysplasia over the past two decades. Our findings reveal several key insights into the field's evolution, structure, and current frontiers:

First, the field has demonstrated substantial growth, particularly since 2015, with research output showing a compound annual growth rate of 18.2%. Publication patterns adhere to established bibliometric distributions, indicating development of a mature research domain despite its relatively recent emergence.
Second, the intellectual structure of the field comprises three distinct knowledge domains—stem cell biology, respiratory physiology, and clinical neonatology—with evidence of suboptimal integration between basic science and clinical research. This translational gap is further evidenced by the temporal lag between preclinical and clinical publications and the relatively small proportion (18.6%) of clinical studies among highly cited papers.
Third, our analysis demonstrates a clear shift in therapeutic focus from whole-cell MSC applications toward MSC-derived extracellular vesicles, particularly since 2018. This trend, identified through keyword burst analysis and citation patterns, reflects the field's refinement toward potentially more translatable cell-free therapeutic approaches.
Fourth, the research landscape shows significant geographical and institutional concentration, with limited representation from regions with high BPD burden. Enhanced international collaboration could improve both the relevance and implementation of research findings globally.
Fifth, emerging keywords indicate expansion of MSC applications beyond pulmonary pathology to other neonatal conditions, suggesting recognition of broader potential benefits of MSC therapies for preterm infants.

These findings provide an evidential basis for directing future research efforts toward: (1) strengthening translational pipelines between preclinical and clinical investigation; (2) standardizing isolation, characterization, and administration protocols for MSC-derived extracellular vesicles; (3) expanding international research collaborations, particularly with developing regions; (4) investigating mechanisms of action through integrated multi-omics approaches; and (5) designing pragmatic clinical trials that address implementation challenges in diverse healthcare settings.

By objectively mapping the structure and evolution of MSC-BPD research, this bibliometric analysis offers valuable insights for researchers, funding agencies, and policymakers seeking to advance this promising therapeutic approach for a devastating neonatal condition.
